# Prognostic significance of serum procalcitonin in patients with unresectable hepatocellular carcinoma treated with transcatheter arterial chemoembolization: A retrospective analysis of 509 cases

**DOI:** 10.1097/MD.0000000000007438

**Published:** 2017-07-14

**Authors:** Hujia Shen, Susu Zheng, Rongxin Chen, Xuejuan Jin, Xin Xu, Chuyu Jing, Jiajia Lin, Juan Zhang, Meixia Zhang, Lan Zhang, Xiaoying Xie, Kun Guo, Zhenggang Ren, Shanshan Lin, Boheng Zhang

**Affiliations:** a Liver Cancer Institute and Zhongshan Hospital; b Department of Epidemiology and Biostatistics, Shanghai Institute of Cardiovascular Diseases, Zhongshan Hospital, Fudan University, Shanghai, P.R. China.

**Keywords:** procalcitonin, prognostic value, transcatheter arterial embolization chemoembolization, unresectable hepatocellular carcinoma

## Abstract

Although procalcitonin (PCT) is a valid marker for early diagnosis of bacterial infections, its accuracy in hepatocellular carcinoma (HCC) patients is unknown. The aim of this study was to investigate the prognostic significance of PCT in patients with unresectable HCC treated with transcatheter arterial chemoembolization (TACE).

A total of 509 patients with unresectable HCC initially treated with TACE were enrolled in this retrospective study. According to quartile of the PCT values, all patients were divided into 4 groups. Overall survival (OS) was evaluated with the Kaplan–Meier method. Significant difference was estimated with the Log rank method. Univariate and multivariate analyses were used for evaluating the significance of the prognostic factor.

The median follow-up period was 18 months and there were significant differences in the survival rates between the 4 groups. The HR (95% CI) for all-cause mortality comparing patients with PCT Quartile2–4 to patient with Quartile1 (HR = 1.00) were 1.353 (1.023–1.791), 1.799 (1.354–2.390), 1.960 (1.455–2.639), respectively, (*P* < .001). PCT level was an important prognostic factor for predicting the prognosis of patients with unresectable HCC treated with TACE.

## 1 Introduction

Hepatocellular carcinoma (HCC) is the second most common cause of tumor-related mortality in China and the third worldwide.^[1,2]^ Resection and liver transplantation are the curative modalities for early HCC; however, most patients do not have chance of radical treatments because of the advanced tumor stages or poor liver function at the initial diagnosis.^[3,4]^

So far transcatheter arterial chemoembolization (TACE) is still one of the most widely used palliative therapies for the unresectable HCC.^[5,6]^ Two randomized trials performed after 2000 showed the survival benefit for the patients with inoperable HCC and the compensated liver function upon receiving TACE treatment.^[7,8]^ At the moment, there is a general consensus that TACE can provide survival benefits in patients with the intermediate HCC [Barcelona Clinic Liver Cancer (BCLC) classification stage B].^[9,10]^

Procalcitonin (PCT) is a precursor of the hormone calcitonin, and its serum concentration in healthy people is <0.01 ng/mL. Serum PCT is raised in bacterial infections, but remains low in viral infections and nonspecific inflammatory diseases. Some studies suggested that serum PCT is interfered by liver function,^[11]^ and it was not an accurate marker of spontaneous bacterial peritonitis in patients with chronic liver disease.^[12]^ Liver is one of the tissues that produce PCT in response to bacterial infections,^[13]^ leading to speculation as to whether PCT levels would be higher in patients with neoplasm in liver. No study has assessed the relationship between serum PCT concentration and HCC.

In this study, we collected 509 HCC cases with BCLC intermediate stage and focused on the prognostic value of pre-TACE serum PCT by analyzing patients’ OS.

## 2 Patients and methods

### 2.1 Patients

The study was conducted as a retrospective analysis of a database that had been prospectively collected at a single clinical center. Data were compiled from 509 consecutive patients with HCC who had been treated with TACE at the Liver Cancer Institute of Zhongshan Hospital of Fudan University between January 2011 and December 2014. Diagnosis was made from American Association for the Study of Liver Disease (AASLD) criteria of HCC. Classical HCC was diagnosed for tumors showing intense arterial uptake of contrast agent followed by washout in the venous-delayed phases, by contrast-enhanced magnetic resonance imaging (MRI) or computed tomography (CT). Patients with poor liver function (Child- Pugh C) were excluded from this study. We also excluded patients with a low peripheral white blood cell count (< 3.0∗ 10^9^/L), or low platelet count (<50∗10^9^/L). Local or systemic infection was also contraindication for TACE.

This study was approved by research ethics committee of Zhongshan Hospital. Informed consent was waived because of the retrospective nature of the study.

### 2.2 Transcatheter arterial chemoembolization procedures

TACE was performed with a conventional procedure. Superior mesenteric and celiac arteriography was performed initially to assess anatomy, tumor staining, and the tumor-feeding artery. The right phrenic artery or other feeding arteries would be inserted for angiograph, in the case of no tumor-supplying branches from the hepatic artery or the superior mesenteric artery. The tumor-feeding arteries were catheterized as close as possible to the tumor, followed by infusion of 1 g of 5-fluorouracil, 100 to 150 mg of oxaliplatin, and then 30 mg of mixed Epirubicin with 5 to 20 mL of lipiodol. The amount of chemotherapeutic agents or lipiodol was determined based on the tumor diameter, liver function, peripheral blood count, and vasculature of the tumor. For some patients with large and hypervascular tumors, gelatin sponge particles were used. TACE was repeated at an interval of 1.5 to 3 months, usually until CT scans and AFP levels suggested stabilization of the tumor.

Liver and renal function tests, full blood count, clotting profile, AFP, and viral hepatitis status were checked before TACE, and the results were entered into a computerized prospective database.

### 2.3 Follow-up

Patients were followed up with an interval of 1 to 3 months. Serum AFP, blood routine, liver function, and enhanced CT or MRI were the routine items for the follow-up after TACE. When tumors were confirmed by typical imaging appearance, patients were treated with appropriate management, including repeated TACE, RFA, surgical resection, percutaneous ethanol injection or radiotherapy.

### 2.4 Statistical analysis

Analysis was performed using SPSS 19.0 for Windows (IBM Corporation, Armonk, NY). Classification data use case number and percentage (%). Quantitative data for normal distribution are expressed as mean (standard deviation), and the quantitative data for skewed distribution are expressed as the median and the quartile range. Differences between 4 groups were analyzed using 1-way ANOVA (normal distribution and homoscedasticity), Kruskal –Wallis rank sum test (skewed distribution or heteroscedasticity). The Student-Newman-Keuls *q* test was used to compare the mean values of multiple samples. The Cochran–Mantel–Haenszel chi square test was used for categorical data. OS was calculated from the date of first TACE procedure to the date of patient's death or the last follow-up. Cumulative survival curves for each variable were obtained by using the Kaplan–Meier method and the differences were compared using the Log rank test. Variables that achieved statistical significance (*P* < .05) in the univariate analysis were subsequently included in a multivariate analysis using a stepwise forward Cox regression procedure to identify factors independently associated with mortality. The Cox proportional hazards model was used to calculate the relative risk of mortality.

## 3 Results

During the study period, there were 509 hospitalizations with the diagnosis of HCC based on AASLD in Liver Oncology Departments. The study cohort had a median age of 57.7 (range 23.0–91.0) years. Overall, 85.3% (434/509) were male and 14.7% (75/509) were female, 98% (499 /509) were positive for hepatitis B virus (HBV) infection, and 35% (178/509) were positive for HBVDNA. On the basis of the Child–Pugh liver function grading, the study group contained 96.3% (490/509) in class A and 3.7% (19/ 509) in class B.

The cohort was followed for a median observation time of 18.0 (range 0.07–88.2) months. During this period 454 of 509 (89.2%) died. Median time to death was 13.2 (range 0.07–73.8) months.

### 3.1 Baseline characteristics of HCC patients across PCT quartiles

Baseline characteristics of the 509 study patients are summarized in Table 1. According to quartile of the PCT values, all patients were divided into 4 groups. The variables tested by univariate analysis were sex, age, tumor size, α-fetoprotein (AFP), total bilirubin (TB), albumin (A), alanine aminotransferase (ALT), aspertate aminotransferase (AST), γ-glutamyl transferase (GGT), prothrombin time (PT), and HBVDNA status. The results indicated that AFP, tumor size, TB, A, ALT, AST, GGT, positive for HBVDNA, were significantly different between the 4 groups (Table 1).

**Table 1:**
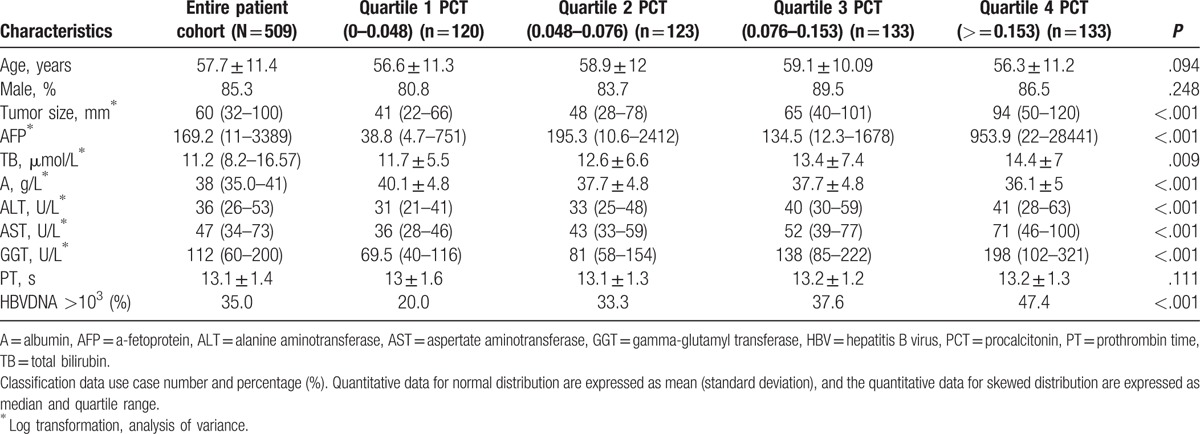
Baseline characteristics, stratified by PCT Status.

### 3.2 Overall mortality

**Figure 1:**
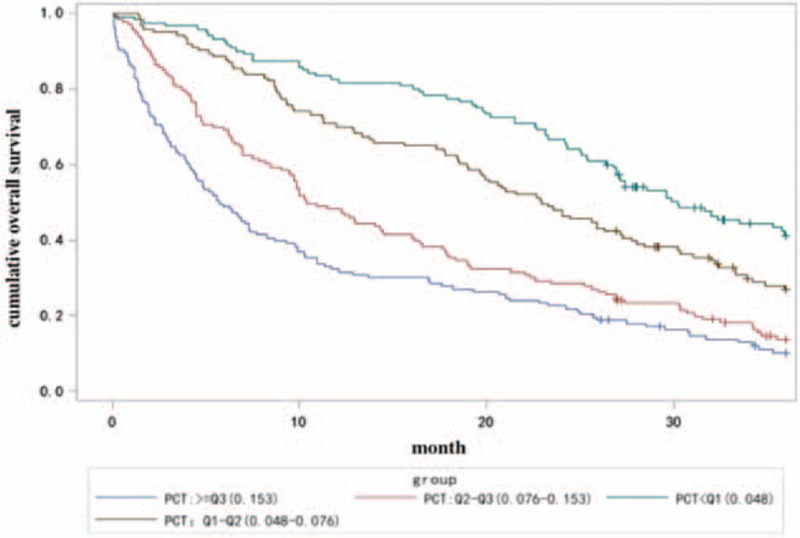
Kaplan–Meier plots for overall mortality.

The median survival time of each PCT group was 28, 23, 10.4, and 5.7months, respectively. Kaplan–Meier graphs for each PCT group are presented in Fig. 1. The survival curve in quartile was significantly, Log rank *P* < .0001.

After a 36 months follow-up, there were significant differences in the survival rate between the 4 groups (Fig. 1). Follow-up to 60 months, the similar trend is still visible. Univariate analyses of PCT demonstrated that higher PCT was associated with poorer OS rates. PCT was an independent risk factor for OS.

### 3.3 The relationship between PCT and tumor size

**Figure 2:**
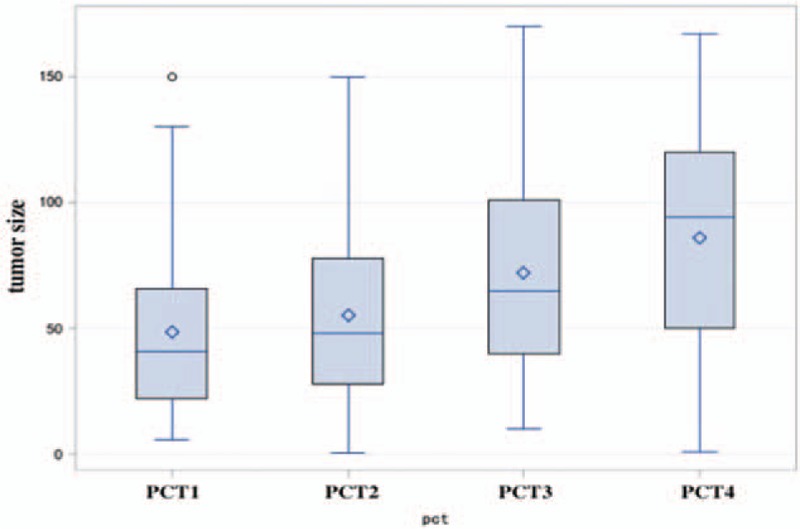
Univariate analyses of PCT demonstrated that PCT was positive correlation with tumor size. PCT = procalcitonin.

The median and quartile range of tumor size in each PCT group was 41 (22–66), 48 (28–78), 65 (40–101), and 94 (50–120) mm (*P* < .001). Univariate analyses demonstrated that PCT was positively correlated with tumor size (Fig. 2).

### 3.4 Multivariate cox analysis

Analysis of prognostic factors affecting OS was performed. High PCT was significantly associated with mortality after adjusted for age, gender, tumor size, AFP, GGT, HBVDNA in multivariate Cox models (Table 2). Other significant risk factors associated with increased mortality were tumor size, AFP, GGT, positive for HBVDNA. PCT, AFP, GGT, tumor size and positive for HBVDNA were found to be independent risk factors of OS (Table 2).

**Table 2:**
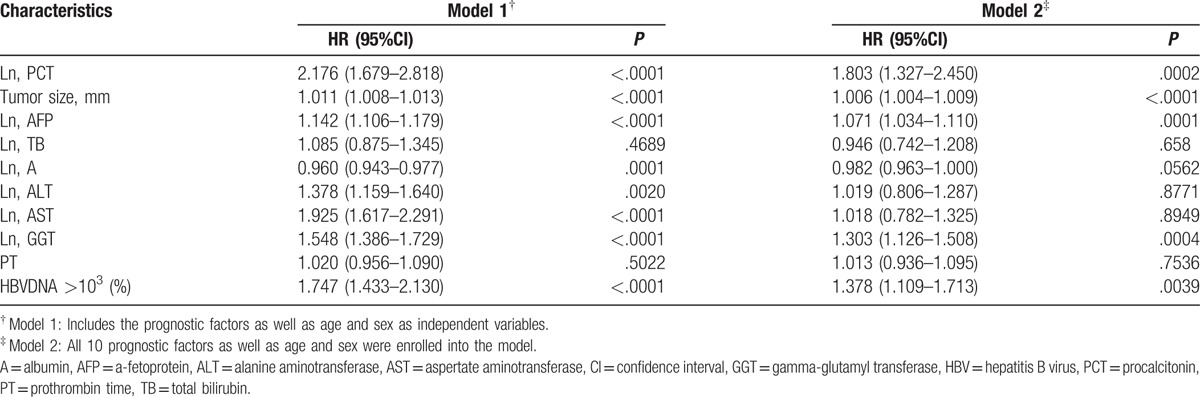
Multivariate proportional hazards regression analysis of biomarker factors associated with overall survival.

There was a strong dose-response relationship between the levels of PCT and the risks of all cause-mortality (Table 3). The HR (95% CI) for all-cause mortality comparing patients with PCT Quartile2–4 to patient with Quartile1 (HR = 1.00) were 1.353 (1.023–1.791), 1.799 (1.354–2.390), and 1.960 (1.455–2.639), respectively, with a *P*-value for linear trend < .001 (*P* for gender interaction > .15, data not shown). The pattern of dose–response associations was similar between the patients with tumor size >80 mm and <80 mm.

**Table 3:**
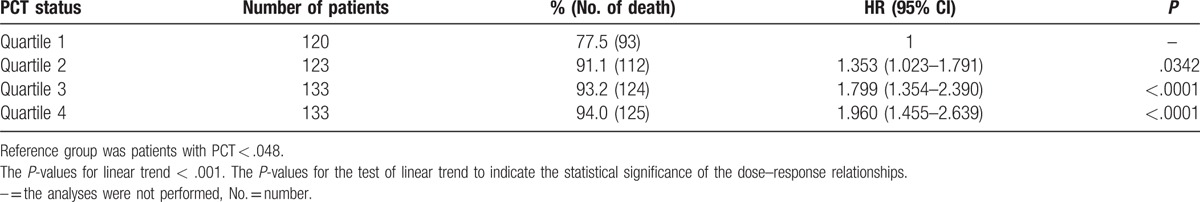
Multivariable adjusted hazard ratios and 95% confidence interval of all cause-mortality associated with the PCT status.

## 4 Discussion

We often see large difference in prognosis among patients with the same stage of BCLC, clinically. The prognostic molecular markers for HCC have been studied extensively, especially those simple serum biochemical markers, such as AFP, GGT, CRP, whose values can be relatively inexpensively and easily obtained in daily clinical practice.

PCT is a biochemical marker that holds great promise for the management of infectious diseases, such as pneumonia, severe sepsis/septic shock, and bloodstream infection.^[14]^Many previous studies have shown that PCT was superior to C-reactive protein, interleukin-6, and leukocyte counts in early diagnosis of bacterial infections in patients without liver disease,^[15–17]^ but its role in hepatocellular carcinoma is still unclear.

At present, the specific production and metabolic pathways of PCT are not clear. However, liver is one of the tissues that produce PCT in response to bacterial infections.^[13]^ Qu et al^[18]^ found PCT had a moderate positive correlation with serum TBIL level in patients with chronic liver disease, inferring the cut-off values for prediction of infections were related to liver function. In our study, the expression level of PCT was positively correlated with tumor diameter, probably due to not only the chronic liver damage in most patients in this study, but also altered expression of PCT in plasma by HCC cells.

Our study demonstrated that the PCT level was associated with the prognosis of patients with unresectable HCC and there was a strong dose–response relationship between the levels of PCT and the risks of all cause-mortality. However, GGT and positive for HBVDNA are also independent prognostic factors according to our statistical analysis. GGT is a stable serum molecule from liver, and high levels of serum GGT are indicative of hepatic or bile tract-associated diseases.^[19]^ The mechanism underlying how HBV targets the hepatocytes, turns over, and excretes out of hepatocytes has been mostly elucidated in recent studies.^[20–23]^ High level of hepatitis B virus replication is directly associated with the development of acute or chronic inflammation, fibrosis, cirrhosis, and HCC.^[24,25]^ So, the expression of PCT is closely related to chronic virus hepatitis and liver function. It can also explain the consistency of PCT, GGT, and HBVDNA in predicting prognosis.

Another possibility of elevated PCT level in HCC patients is tumor-associated inflammatory response.^[26–29]^ The presence of pro-inflammatory cytokines, which are produced by tumor necrosis or local tissue damage, may further stimulate the systemic inflammatory response and be largely involved in neoplastic progression.^[30,31]^

The use of prophylactic antibiotics following TACE is controversial. Dabbous et al^[32]^ reported PCT was a promising marker for diagnosis of sepsis in HCC patients treated by TACE and PCT levels were significant correlated with positive bacterial cultures and post interventional CRP. The early detection of bacterial infections and HCC could ameliorate the prognosis of cirrhosis.^[33]^ Therefore, aside from PCT, clinical manifestations, radiological images and other laboratory evidences, such as C-reactive protein, leukocyte counts, blood culture are all needed in early diagnosis of bacterial infections in HCC patients. Several novel biomarkers such as lypopolysaccharide binding-protein, mid-regional fragment of pro-adrenomedullin and delta neutrophil index may become promising indicators of early diagnosis of bacterial infections in HCC patients.^[33–35]^

There are some limitations to our study. First, for majority of the cases pathological examination was not carried out. Hence, not only the degree of malignancy, but also that of liver fibrosis remains unclear. Moreover, there is no further stratification analysis for accurate cut-off point for identifying infections in HCC patients.

A further study would be to quantify the PCT plasma levels that actually alert physicians on explicit bacterial infections in HCC patients. Moreover, the regulatory mechanism of PCT level in patients with chronic liver disease needs further research.

## 5 Conclusion

The PCT level was an important prognostic factor for predicting the prognosis of patients with unresctable HCC treated with TACE.
